# “How the other half live”: Lay perspectives on health inequalities in an age of austerity

**DOI:** 10.1016/j.socscimed.2017.05.021

**Published:** 2017-08

**Authors:** Kayleigh Garthwaite, Clare Bambra

**Affiliations:** Institute of Health & Society, Newcastle University, Baddiley-Clark Building, Richardson Road, NE2 4AX United Kingdom

**Keywords:** United Kingdom, Health inequalities, Lay perspectives, Fatalism, Ethnography, Austerity, Behaviour

## Abstract

This paper examines how people living in two socially contrasting areas of Stockton on Tees, North East England experience, explain, and understand the stark health inequalities in their town. Participants displayed opinions that fluctuated between a variety of converging and contrasting explanations. Three years of ethnographic observation in both areas (2014–2017) generated explanations which initially focused closely on behavioural and individualised factors, whilst 118 qualitative interviews subsequently revealed more nuanced justifications, which prioritised more structural, material and psychosocial influences. Findings indicate that inequalities in healthcare, including access, the importance of judgemental attitudes, and perceived place stigma, would then be offered as explanations for the stark gap in spatial inequalities in the area. Notions of fatalism, linked to (a lack of) choice, control, and fear of the future, were common reasons given for inequalities across all participants. We conclude by arguing for a prioritisation of listening to, and working to understand, the experiences of communities experiencing the brunt of health inequalities; especially important at a time of austerity.

## Highlights

• Unique insights into how people in socially contrasting areas understand health inequalities.
• Uses detailed ethnographic methodologies over a three-year period.
• Reveals how judgemental attitudes can impact on health.
• Argues for a deeper understanding of complexity in communities.

## 1 Introduction

Considerable research attention has been paid to identify and explain how health and place interrelate, and the resultant impact upon health inequalities (Bambra, 2016; Bernard et al., 2007; Curtis and Rees Jones, 1998, Macintyre et al., 2002, Sloggett and Joshi, 1994; amongst others). Geographical research has been dominated by the debate between compositional (population characteristics of people living in particular areas including demographic, health behaviours and individual-level socio-economic status) and contextual (area-level factors including the social, economic and physical environment) explanations. This academic debate - about the causes and complexities of geographical inequalities in health – could benefit from lay perspectives on health and place and the causes of health inequalities particularly from people living in the most and least deprived communities.

To date, research by Popay et al., 2003, Macintyre et al., 2005 and Davidson et al., 2006, Davidson et al., 2008 has examined lay perspectives in socio-economically contrasting areas of cities across northern England and Scotland. Other studies (such as Blaxter, 1983, Blaxter, 1997, Parry et al., 2007, Mackenzie et al., 2016) have examined the perspectives of people living in the most deprived areas. These studies have employed mixed methodologies, including surveys, focus groups, and in-depth interviews; ethnographic research which explores the everyday lived realities of health inequalities is notably absent. Davidson et al. (2008: 168) have recognised this gap in the literature, and noted how “even fewer studies have specifically focused on the relationships between the types of place people reside in, and their experiences of, and attitudes to, health inequalities”. Mackenzie et al. (2016) evidence not only material factors, but also explanations of the interplay between power and politics, with an explicit focus on how behavioural explanations can be integrated into such explanations.

This paper, in keeping with Popay (Popay et al., 2003), Macintyre (Macintyre et al., 2005), and Davidson et al., 2006, Davidson et al., 2008, directly explores the lived experience of- and perspectives on-geographical inequalities in health of people from socio-economically contrasting areas. Following Backett (1992: 257) in her research into lay health moralities in middle class families, the key purpose of this study was to “develop understandings of how beliefs and behaviours which may have implications for health are part of the fabric of daily life”. In particular, this study focused upon people's everyday awareness and understanding of living in a place with severe health inequalities, and to question how this might be affected at a time of austerity. As such, it is one of the first studies to examine lay perspectives on health inequalities during austerity.

### 1.1 Geographical inequalities in health

Neighbourhoods that are the most deprived have worse health than those that are less deprived – and this follows a spatial gradient with each increase in deprivation resulting in a decrease in average health. In England, the gap between the most and least deprived areas is 9 years average life expectancy for men and around 7 years for women. As noted earlier, geographical research has tried to explain these differences through looking at compositional and contextual factors – and their interaction (Cummins et al., 2007).

The compositional explanation asserts that the health of a given area is the result of the characteristics of the people who live there in terms of demographic [age, sex and ethnicity], health-related behaviours [smoking, alcohol, physical activity, diet, drugs] and socio-economic [income, education, occupation]) factors. There is an extensive literature linking socio-economic status to health: people with higher occupational status (e.g. professionals such as teachers or lawyers), education or income have better health outcomes than non-professional workers (e.g. manual workers), or those with lower levels of education or income. Health follows a social gradient – the higher the social position, the better the health. Health inequality is therefore not an issue just of poverty, but is related to economic inequality more widely (Wilkinson and Pickett, 2010).

The literature suggests that there are several interacting pathways linking individual-level socio-economic status and health: behavioural, material, psychosocial, and life course (Bartley, 2004). The ‘materialist’ explanation argues that it is income-levels and what a decent or high income enables compared to a lower one such as access to health-benefitting goods and services (e.g. health care access, schools, transport, social care) and limiting exposures to particular material risk factors (e.g. poor housing, inadequate diet, physical hazards at work, environmental exposures). The ‘behavioural-cultural’ theory asserts that the causal mechanisms are higher rates of health-damaging behaviours in lower socio-economic groups – which may be more culturally acceptable amongst lower socio-economic groups. The ‘psychosocial’ explanation focuses on the adverse biological consequences of psychological and social domination and subordination, superiority and inferiority. The ‘life course’ approach combines aspects of the other explanations, thereby allowing different causal mechanisms and processes to explain the social gradient in different diseases. It also highlights the role of the accumulation of disadvantage over the ‘life course’ – combining the amount of time different people have spent in more/less disadvantaged circumstances.

The contextual approach instead focuses on the health effects of the economic, social, physical and political environment of a place, arguing that regardless of individual factors, where you live also matters (Bambra, 2016). Health promoting environments are more likely to be found in affluent as compared to deprived areas. Area-economic factors that influence health include area poverty rates, unemployment rates, wages, and types of employment in the area. Social place-based factors include opportunity structures and collective social functioning and practices - the services provided (publicly or privately) to support people in their daily lives as well as the reputation and history of an area as well as local cultures (Macintyre et al., 2002). In terms of the physical environment, there is a sizeable literature, for instance, on the positive health effects of access to green space (Mitchell and Popham, 2007), as well as the negative health effects of brownfield or contaminated land (Bambra et al., 2014) as well as air pollution (Stafford and McCarthy, 2006) or neighbourhood regeneration (Egan et al., 2015). Compositional and contextual factors though are not separate phenomena: they interact and shape one another (Cummins et al., 2007).

This paper examines whether and how lay perspectives reflect these contextual and compositional theories of geographical inequalities in health.

### 1.2 Austerity and health inequalities

Although spatial inequalities in health within the UK have been much discussed, there is less empirical assessment of the effects of the current programme of austerity on these inequalities (Pearce, 2013) - manifested as large-scale cuts to central and local government budgets, as well as an NHS funding freeze and cuts to welfare services and benefits (Bambra and Garthwaite, 2015). However, recent (primarily quantitative) research has indicated that austerity and welfare reforms are having adverse effects on the most vulnerable in society. In their study on self-harm, Barnes et al. (2016:1) reported that “economic hardships resulting from the recession and austerity measures accumulated or acted as a ‘final straw’ to trigger self-harm”. They emphasised that “changes in welfare benefits may have contributed” to this rise (2016: 132). Niedzwiedz et al. (2016) found that reductions in spending levels or increased conditionality may adversely affect the mental health of disadvantaged social groups. Highest levels of foodbank use have occurred where there have been the highest rates of benefit sanctioning, unemployment, and cuts in central welfare spending (Loopstra et al., 2015).

Accompanying austerity measures and ongoing reforms to the social security system is a dominant narrative that characterises people living on a low income as ‘feckless’, lacking in aspiration, and engaging in poor lifestyle choices (Garthwaite, 2016a), which can lead to stigma, a worsening of already poor mental health and the risk of widening health inequalities (Hatzenbuehler et al., 2013, Scambler, 2006). Pearce (2013: 1922) has emphasised an (arguably surprising) lack of attention on this issue from a geographical perspective:

“whilst there is a voluminous literature evaluating the role of various forms of discrimination in understanding health and inequalities, geographical accounts of discrimination have been thin on the ground.”

Pearce observes how austerity measures evident in the UK, as well as other European countries, will undoubtedly have implications for health inequalities which are yet to be experienced or documented. Making a link between this process and stigma production, Pearce argues that “one of the likely implications of reducing investment into communities with a multitude of social problems is that such places will become increasingly stigmatised, which is likely to be detrimental to the health of local residents” (2013: 1924). This research project is situated at this nexus, and the findings reported here will draw attention to the relationship between place, health inequalities, and austerity through the lay perspectives of those living in the most and least deprived areas of Stockton-on-Tees, the North East town with the largest health inequalities in England. This paper will therefore add to the health inequalities literature in terms of strengthening our understanding of lay perspectives and their relationship with existing theories, whilst being one of the first studies to examine lay perspectives in a period of austerity.

### 1.3 Lay perspectives

The importance of lay knowledge has emerged as being central to knowledge and understanding surrounding health inequalities (Backett-Milburn et al., 2003, Blaxter, 1997, Davidson et al., 2006, Davidson et al., 2008, Elliott et al., 2015, Macintyre et al., 2005, Popay et al., 1998, Popay et al., 2003 amongst others). As Davidson et al. (2008: 1368) and others (Blaxter, 1997, Backett-Milburn et al., 2003, Macintyre et al., 2005) have recognised, very few studies have directly explored lay understandings of the causes of health inequalities in general. Further, there has been even less attention dedicated to exploring the relationships between where people live, and their experiences of - and attitudes towards - health inequalities, with a focus on lay understandings of health inequalities at a time of austerity being relatively absent (see Mackenzie et al., 2016 for a notable exception). Ethnographic methods have also been underutilised in this regard.

Using both quantitative and qualitative methodology, Popay et al. (2003) studied the views of people living in cities in the North West of England. The survey findings showed how people living in disadvantaged areas offered explanations which included both individualistic and structural factors, including an emphasis on the importance of ‘place’. In contrast, findings from their qualitative interviews found people living in disadvantaged areas were reluctant to accept the notion of health inequalities between areas and social groups (Popay et al., 2003: 1). Macintyre et al. (2005: 314) reported similar findings from analysis of a longitudinal postal survey in the West of Scotland. The studies by Blaxter, 1983, Blaxter, 1997 also found that people living in deprived areas were less likely to acknowledge the links between health and place and less likely to view the causes as material or environmental. Davidson et al. (2008) though found a greater acceptance of the existence of health inequalities. In terms of pathways, a recent review of 25 studies of lay understandings of area-level deprivation and health (Smith, 2017) found that there is evidence of behavioural, material, and psychosocial factors in lay understandings of health and place. Several studies in this review found a clear understanding of the links between income, unemployment and health; other studies found that participants privileged behavioural factors (particularly smoking and alcohol) or environmental ones such as housing, crime, transport or services. The review though concluded that psychosocial pathways were the most prominent explanations linking area-level deprivation to mental health particularly social cohesion and local pride.

## 2 Study design and methods

This article draws on data from ‘Health Inequalities in an Age of Austerity: the Stockton-on-Tees study’, a five year, mixed methods project examining localised health inequalities in an era of austerity in the town of Stockton-on-Tees, North East England. Stockton-on-Tees, has the highest geographical health inequalities within a single local authority in England both for men (at a 17.3 year difference in life expectancy at birth) and for women (11.4 year gap in life expectancy) (Public Health England, 2015). Stockton-on-Tees has a population of 191,600 residents (Census, 2011) and features high levels of social inequality, with some areas of the local authority having low levels of deprivation (e.g. Hartburn; Ingleby Barwick; Yarm) and others nearby characterised by high levels of deprivation (e.g. Town Centre; Hardwick; Port Clarence).

This article uses ethnographic methods and qualitative interviews. Studying health inequalities through ethnography allows people's lived experiences to be studied in everyday contexts, following a flexible research design, with participant observation and relatively informal conversations forming a central part of the research process. Undertaking participant observation of a particular place involves the researcher walking or driving through local places to observe social environments and happenings (Pink et al., 2010: 3). As such, routine daily activities across the two field sites were observed in public places that made seeking informed consent unfeasible. The researcher's casual conversations with residents in local places were included as non-verbatim data in observation notes.

The Town Centre ward is the most deprived in the borough, and is the 17th most deprived ward in England. The ward particularly experiences health, disability, and employment deprivation. 27.1% of economically active people are unemployed, and 10.2% are receiving out of work benefits. Only 22% of residents own a house; this is significantly lower than the borough average of 69%. The majority (53%) live in socially rented accommodation and 23% live in private rented accommodation. In the 2011 Census, 12% of people reported that they were in bad or very bad health, much higher than the borough average of 6.3%. 26.5% of people have a long-term health problem or disability, this is higher than the borough average of 19.0%. Poor quality housing, takeaway shops, convenience stores selling low quality food, betting and pawn shops, and a pub where all drinks cost £1, are all plentiful in the most deprived area. There has, however, been a recent £38 million regeneration of the High Street, which has been much discussed by participants throughout the research. Fieldwork in the Town Centre ward began in November 2013, with participant observation and interviews carried out in a Trussell Trust foodbank (Garthwaite, 2016a), Citizen's Advice Bureau, children and family centres, community centres, gardening clubs, cafes, and coffee mornings, alongside engagement with charities, events and services in the area.

From March 2014, participant observation began in Hartburn, the third least deprived out of the 26 in the borough, and one of the least deprived wards in England. The unemployment rate here is 5.1%, lower than the average for England and Wales of 7.6%, and the Stockton-on-Tees average of 9.6%. Only 1.2% of people in the Hartburn ward are receiving out of work benefits. 92% of residents own a house outright or are buying it with a mortgage; only 1% live in socially rented accommodation and 6% live in private rented accommodation, both are much lower than the borough averages of 17% and 13% respectively. 4.3% of people reported that they were in bad or very bad health, this is lower than the borough average of 6.3%, and 19.2% of people have a long-term health problem or disability. The area is characterised by manicured green space, flower beds, attractive period houses and independent businesses such as a delicatessen, a dog grooming parlour, and a florists. Observations and interviews here took place at coffee mornings, yoga classes, cafes, churches, mother and toddler meetings, a credit union, and community centres.

118 qualitative interviews were completed across both areas between 2014 and 2017, alongside detailed participant observation, field notes, documentary research, and photographic data. To ensure a varied sample, in-depth interview participants were sampled across these locations to include variation in age, gender, occupation, marital status. Participants were recruited by a mix of approaches – they were asked following ethnographic observation, and sometimes acted as gatekeepers with snowballing approaches used to recruit others. Topics covered during the in-depth interviews included (but were not limited to): area perceptions; health and health inequalities; austerity and welfare reform; social networks; community; employment; and social security benefits receipt. Interviews that were arranged to take place in people's homes were recorded and transcribed verbatim. The age range of the overall sample varied from 16 to 78 years old, and was almost equally split in terms of men and women. Ethnographic observations captured a wider age range.

Participation was voluntary, confidential, and secured by either verbal or written informed consent where possible. Interviews were transcribed verbatim and the transcripts produced included references to both field notes made and photographs taken. Data were fully anonymised before transcripts were analysed thematically, using open coding to identify initial categories. Data was then further broken down into sub-themes, allowing us to compare and contrast data in a detailed manner. In this way, thematic content analysis was used to analyse the data and extract relevant relationships between study ethnographic observation and interview results. In this way participants’ verbal accounts and non-verbal behaviours could be analysed and coded in one dataset to give a fuller picture. NVivo 10 software was used to facilitate and organise the analysis. The research was approved in advance by the Durham University Department of Geography Ethics Committee.

## 3 Findings

### 3.1 Converging and contrasting explanations

Conversations and observations in both the most and least deprived areas generated explanations which initially focused closely on behaviour and individualised factors, suggesting smoking, alcohol, and the consumption of unhealthy food were root causes of the gap in life expectancy within their area. In-depth interviews revealed more nuanced justifications, for both groups, which prioritised altogether more structural, material and psychosocial factors, such as income, housing, happiness, and community networks. These categories were neither separate nor distinct, and participants often displayed opinions that fluctuated between a variety of explanations. Following Macintyre (1997: 728), participants distinguished between ‘hard’ explanations for inequalities – in other words, differences were completely accounted for by health damaging behaviours (smoking, poor diet, inappropriate use of health services etc.) – and ‘soft’ explanations, which believe certain health damaging behaviours have a social class gradient which contributes to the gradient in ill health and early death.

Explanations that centred on behaviour and education were mostly found in the perspectives of people in the least deprived area, and particularly during ethnographic observation. This tended to be linked to the transmission of generational family values. Katie, 41, worked in marketing and lived in the more affluent suburb with her husband and two children. Katie placed an emphasis on the importance of cultural values and aspirations of education, but also accepts that the “odds are stacked against you” if you're living in one of the most deprived areas:

“You're talking a lot about [a] third generation of people who've never had a job. You learn from your parents, you learn your principles and values and everything. Everyone's looking for the fast and easy way round everything, it's just not realistic and they just forget about education. It goes right back to even at the beginning, if you're in a shit school and there's people with all different needs, the odds are stacked against you, and then if you're feeding your kids crap right at the beginning, it's like what's going on? So I can see why people aren't living longer, and like the smoking thing, I mean I've smoked and as soon as I found out I was pregnant I stopped, and now I wouldn't dream of it. But I suppose if you live where everybody is smoking around you, it's just what you do, isn't it?”

The following field notes extract identifies how fieldwork observations and conversations tended to centre on ‘hard’ (Macintyre) behavioural explanations:

**Field notes**

**9th June 2015**

It's my first day of the credit union that's been set up by some of the people I've come to know in Hartburn. Heather invited me into the back room for a coffee and offered to introduce me to the others who I don't know, who are looking fairly suspicious of me to be honest. I get sat next to a serious looking woman, Jennifer, and Kathyrn comes to join us and starts explaining about the project. Jennifer looked at me as if I was stupid and simply said: ‘Well it's all about behaviour, isn't it?’ She seemed horrified that a £1 million grant was being used to investigate something that she believed could be explained away by faulty behaviour. I said obviously behaviour is part of the whole story, but actually isn't the major factor in the gap in life expectancy according to our survey findings – income, education, housing and quite frankly money are more important. She doesn't look convinced: ‘I would imagine behaviour is the most important’ she said, and turned around to talk to someone else.

In contrast to findings from Popay et al. (2003) and Macintyre et al. (2005) though, participants in this study living in the most deprived areas also recognised that income, housing, and stress were all factors in explaining the severe health inequalities in Stockton-on-Tees. Glen, a chef working on a zero hours’ contract, lived in a deprived area a couple of miles outside of the Town Centre. He believed the gap in life expectancy was linked to lower stress and higher income levels in the more affluent areas of town. Despite this, he also linked the difference to the behaviour and lifestyles of people living in the most deprived areas:

“I think it's cos them in Hartburn have jobs and they have loads of money. They've got good work and they've got good living. And I think some of these in the Town Centre they just go around getting drunk, being homeless. It's a lifestyle choice, it gets them out of it for a couple of days, y'know?”

Participants from the more-deprived areas in Davidson et al.'s (2008) study discussed how deprivation was ‘written in the body’ in terms of premature ageing. Our findings show that participants across both the most and least deprived areas recognised how poverty can impact upon peoples' health and bodies, physically and mentally. In an in-depth interview with Steph, 42, a welfare rights adviser who lived in one of the least deprived areas, she expressed her “shock” at how the combination of multiple traumatic incidences, such as bereavement, sexual abuse, domestic violence, and ill health, can impact upon people physically:

“I suppose you know anecdotally which areas have more concentrations of poor health but sometimes it shocks me how much it ages people. I think they look old, you can see it in their faces the way they are and I think that's sad. They tell me their date of birth and I think ‘God you're my age’, or a few years older and I think what is it that's so different about us, that we look so different? But then a lot of people I deal with, they've had not just one kind of traumatic thing happen to them, they might have had 2 or 3 things that would be almost kind of nobody I know in my friendship circle has had that happen to them, but that person has had like multiple”.

Living in the least deprived area, Catherine, 65, initially spoke during ethnographic encounters of how smoking and obesity were key factors in explaining the large gap in life expectancy, but during an in-depth interview she also identified how psychosocial factors can play a role in explaining the health inequalities within the area:

“Smoking, obesity … but the overriding thing is that they don't seem happy. I really would say that the most notable thing is they're not going round with big smiles on their face, happy jolly people. They're miserable. So that would come back to the mental health issues, wouldn't it? It would wear you down”.

The impact of these cumulative traumas can then further widen pre-existing health inequalities, and perhaps go some way to understanding ideas of fatalism, choice and hope that participants felt helped to explain the gap in life expectancy in Stockton-on-Tees.

### 3.2 Fatalism, choice and opportunity in a time of austerity

Notions of fatalism, linked to (a lack of) choice, control, and opportunity, were common reasons given for inequalities across all participants, but more often from those living in the least deprived areas. Heather, 72, a trustee of various mental health and addiction charities, felt fatalism was key to explaining the gap in life expectancy within the borough:

“People feel stuck, don't they? People don't feel that any effort they make is going to make a difference where out there in the affluent areas we know that efforts we make will make a difference. There's an element … it isn't so much confidence, well it is confidence but it's also a bit fatalistic, ‘Well whatever I'm doing doesn't matter’, and I think that's why people don't bother with healthy living ‘Well do I want to be here?’ y'know if you've developed lots and lots of poor health, what's the joy of living to 100? And I guess, but I don't know, I guess it's a sense of ‘Well whatever I do won't make a difference’ for myself or anyone else. It's about drive isn't it, and have they ever had drive in the Town Centre? Because people who have drive have got out.”

Heather strongly associated this sense of fatalism with the geographical boundaries of the Town Centre and the perceived culture amongst people living there that combined to create an overall sense of hopelessness or lack of control. Carol, 68, was a former health visitor who worked within deprived communities nearby for over 40 years. Living in the least deprived area, Carol agreed that difficulties in thinking about the future may lead to “impulsive behaviour” which she defined as drug taking and smoking:

“When you've got this impulsive behaviour, not thinking about tomorrow then you don't care very much about the future of your health, either. You're thinking about today. And a lot of these people who I worked with, who aren't going to live very long, actually just getting through today, and they don't care about 20 years' time or 10 years' time. Sometimes today is so awful for them”.

Here, Carol recognises that everyday life can be filled with multiple and complex issues, making it impossible to plan and even imagine a future. Carol, and others across both in-depth interviews and ethnographic observation, regularly referred to the notion of ‘luck’ for helping to explain the differences between their situation and those living in the most deprived area. “We're kind of a lucky generation really, I think” was often offered as a justification, generally from the older participants who were now retired. Luck was also used as an explanation for the good health that people in the least deprived area experienced.

Everyday worry and hardship was a common theme found in the experiences of people living through austerity in the most deprived area. Simon, 52, was a volunteer at the foodbank after using it three times himself. Currently unemployed, he described the daily struggles he had in making his Employment and Support Allowance of £146.20 per fortnight cover his bills, debt, and food expenditure:

“I get a bit bag of spuds for £2.75 and that lasts for two weeks, if you've got potatoes you can always have chips. Beans, tomatoes is a good one, buy spices every week then you can mix things together. I've had pasta and beans before with spices, mix it in, it's not the best of things to be eating but at least it's a meal. Porridge is good cos with porridge you don't need milk, milk is a luxury. Things like that, just things that'll spread.”

Lauren, 33, received Carer's Allowance for her two sons. She described the difficulty in being able to plan for the future when receiving social security benefits, and how this was influenced by austerity-led welfare reform:

“It's pretty miserable really, I try not to think about the future cos when you get benefits … I sit and watch every Budget in a panic, I get upset the night before, I read all these reports and I think ‘What they gonna take next?’ My whole life is in the balance of the decisions that politicians make, and it's scary and I know it's sort of like that for everybody but it really does feel like that in a big way for us”.

For people living in the least deprived area, conversations would focus on the regular trips to the theatre, language courses, horse riding, ukulele classes, dining out, and frequent holidays. In contrast, people living in the most deprived area often tried to find free things to do, such as go for a walk with their children in the local park, or sit in the High Street on a sunny day, watching the water fountains that had recently been installed as part of a £38 million regeneration of the Town Centre. People across the least deprived area made full use of the local groups and activities that were often free to access, including ones specifically aimed at people who lived in the most deprived areas, such as Sure Start. Heather, speaking about the weekly coffee morning she helps to run, recognised this as being a particular factor in explaining the gap in life expectancy in the area:

“I think people, well you know the people come here, particularly as helpers, they're very active, y'know, walking groups, the community choir, volunteering, the gym. And there are things like cycling groups and walking groups that are provided by the council, but they tend to be taken up by folk like us. We fill them up.”

Ethnographic observations were carried out across various clubs, groups and initiatives aimed at improving the health and wellbeing of people living in the most deprived areas. Due to cuts to local authority budgets, several services and clubs that the researcher became involved with had to be closed due to funding constraints – for instance, a weekly walking group was forced to cease a few months after the researcher joined. Often, such groups would be poorly attended; a notable example being a credit union set up by those living in the least deprived area, but situated within a church on one of the most deprived streets in the town. To date, only members of the congregation had signed up to use it, and there was a sense of frustration and incomprehension as to why people living on the doorstep were not engaging with it. But in spending time in the most deprived area, it became clear that one possible explanation for the reluctance to engage with services such as the credit union was a perceived sense of being subject to judgement and stigma.

### 3.3 The importance of judgement and attitude

Participants in the most deprived areas described a hardening of attitudes towards people living in low income areas. Living in the most deprived area, Lauren, 33, identified the struggles she had with judgemental attitudes, and the effect this could have upon accessing support:

“I think for me it's people's attitudes when you go and seek help. I think if you've never experienced it, someone looking down their nose at you because you don't work, or you've got depression whatever if may be, because you need something from society, you know financially or medically. You'd think nurses and doctors and receptionists, you'd think they'd be nice to you but what I found, and I would say … I would assume that people who are on the bad end of the health gap may feel the same, is that people's attitudes towards you are awful, it makes you not want to ask for help. All the time this message is that you're bad, it's on the telly, you're not a worthy person so at what point do you not access things because you feel it yourself?”

For Lauren, these attitudes were strongly linked to so-called ‘poverty porn’ television programmes which depict a certain lifestyle of benefits receipt or living on a low income. 'Poverty porn' has ‘been used to critique documentary television in post-recession Britain which focuses on people in poverty as a-political diversionary entertainment’ (Jensen, 2014: 2.6). This genre of television depicts people as lazy, criminal, violent, undisciplined and shameless, playing into the media and government rhetoric around people living on a low income. The impact of ‘poverty porn’ is particularly relevant given the second series of Love Productions' *Benefits Street* was set on Kingston Road in the deprived Portrack and Tilery ward, next to the Town Centre. The significance of “not speaking the same cultural language” as patients and the potential resultant impact on health inequalities was reinforced by the Town Centre GP Dr Harrison who spoke of the importance of the health service:

“I can give you the example of doctors, you know you've come into a fancy building here, which is intimidating for people. I speak with a posh voice, I'm wearing a tie which puts a certain barrier up. But also many doctors live in the wealthier parts of town. So they live in this very precious enclave, they drive in in a car, [they're] protected sealed in with air conditioning, they're listening to Radio 4, they're parking it, coming into their own safety environment which is very different to the environment people live in, they're seeing people, then they're going home. Occasionally they might do a home visit but they're not using … they're not really understanding where people are coming from, they will never really understand the financial constraints on people and it isn't just doctors, it's nurses, its health visitors, it's midwives, it's the whole health infrastructure, it's receptionists as well and they can often act as a barrier. We might say ‘How on earth do they still continue to smoke? Don't they know it's bad for them? I've invited them three times to come and they haven't come’ so you get these kind of … these attitudes, and then of course you get organisational culture where people talk about it in the tearoom and it reinforces those attitudes and you then get a kind of ‘them’ and ‘us’, patient blaming culture and it widens health inequalities.”

The attitudes described by Dr Harrison have become progressively more noticeable amidst ongoing austerity and reforms to the social security system, he felt, and it was clear in the perspectives of those living in the most deprived area that they agreed with this. Naomi, 36, a recovering heroin addict, had a range of physical and mental health problems, including gastrointestinal issues, depression and anxiety. Naomi identified a stigmatising and judgemental attitude attached to her accessing the local pharmacy for her methadone:

“Every day I go to the chemist and it's supervised, I have to drink it. In the Stockton area everyone knows what you're going in for, no matter how well you're dressed, they still know what you're going in for so you get the funny looks. People look at you up and down and you know what they're thinking and that gets you down”.

The health implications are clear, with such attitudes possibly impacting upon mental health and wellbeing, as Naomi suggests.

## 4 Discussion and conclusion

This study has outlined lay perspectives on the experiences, understandings and explanations of health inequalities in two geographically close but socio-economically distant areas of a post-industrial town in the North East of England. In terms of explanations, it has found that inequalities in healthcare, including access, the importance of judgemental attitudes, and perceived place stigma, were the most prominent. Researching lay perspectives on health inequalities ethnographically allowed for an understanding of the nuanced and flexible explanations offered for gaps in health and life expectancy. In a review of the data surrounding lay knowledge and perspectives, Blaxter (1997: 750) recognised the complexities and fluidity in seeking lay understandings of health inequalities:

“Throughout the research evidence, lay respondents tend rather to move back and forwards between concepts of cause which seem opposed, but which individuals can keep in equilibrium - belief in responsibility about health behaviour and the importance of healthy mental attitudes on the one hand, and concepts of chance, luck, and inevitability on the other.”

In keeping with findings from Popay et al. (2003) and Macintyre et al. (2005), our results found that explanations for health inequalities varied depending on which method was employed. Findings demonstrate both converging and contrasting explanations for variation in health inequalities. Davidson et al. (2008: 178) found that higher income groups were more likely to question rather than accept statistics on area inequalities in health, and to view faulty health behaviours as the result of lack of education or an irresponsible attitude. In this study, people living across the most and least deprived areas adhered to this perspective, at times, but were also able to attribute health inequalities to material and psychosocial factors. Ethnographic observation generated explanations which initially focused closely on behaviour and individualised factors – ’hard’ explanations (Macintyre, 1997), whilst qualitative interviews revealed more nuanced justifications, for both groups, which prioritised altogether more structural, material and psychosocial influences – the ‘soft’ explanations as put forward by Macintyre (1997). Inequalities in healthcare, including access, the importance of judgemental attitudes, and perceived stigma, were then offered as explanations for the stark gap in spatial inequalities in the area. These lay perspectives link to the wider academic literature on health and place with compositional factors privileged over contextual ones.

A notable difference between the previous work by Popay et al., 2003, Macintyre et al., 2005 and Davidson et al. (2008) and this study is the context of austerity measures and cuts to the social security safety net which do not affect all groups or neighbourhoods equally. The importance of this context is evident when we consider the deeply divisive rhetoric between ‘shirkers’, ‘skivers’, ‘workers’ and ‘scroungers’ being applied to people living on a low income (Garthwaite, 2011) and the emergence of a ‘new welfare commonsense’ as identified by Jensen (2014). The idea of ‘commonsense’ relies heavily on the welfare dependent and deceptive benefit ‘scrounger’ who is then portrayed as a figure of social disgust by politicians and the media. This thereby enables the state to retreat from providing basic levels of welfare support with reliance on charity becoming the norm for many in the most deprived neighbourhoods (Garthwaite, 2016b). As our findings have shown, the resultant judgemental attitudes towards people living in low income areas can then impact negatively upon people's (often already poor) mental health, with the ensuing stigma preventing them from seeking further help and support. Lay accounts of health inequalities across both areas were shaped by oft-repeated stereotypes that focused on individual behaviour and lifestyle choice (hard explanations), which at times obscured material and psychosocial explanations (and their political determinants, such as austerity) for wider societal inequality and in a way let those living in less deprived areas “off the hook” in terms of any shared responsibility.

In both in-depth interviews and ethnographic observations, fatalism, control and opportunity were discussed by participants across both neighbourhoods, but particularly in the least deprived area. This advances the lay perspectives literature as whilst it is in keeping with the importance of psychosocial factors as identified by Smith (2017), it locates a new specific psychosocial pathway expressed by participants. Within the health inequalities literature, the concept of fatalism has been used to explain the supposed unhealthy lifestyles of people living in the most deprived groups (Bolam et al., 2004; Marmot and Wilkinson, 2001). In a study of perspectives on health in middle class families, Backett (1987, cited in Backett, 1992: 264) found respondents were not only more openly fatalistic about health matters but also regularly focused on the wider socio-economic and political factors which they described as constraining an individual's possibilities to 'achieve'. The recurrent phrase 'we're very lucky’ was also evident in Backett's research.

Fatalism has been conceptualized by Savage et al. (2013: 1217) in three different ways - low control over health improvement; low control over lifestyle change; and a fear of change and the unexpected. The most relevant to the findings presented here is low control over health improvement, which was linked by participants to the negative effects that living a life affected by multiple and complex issues; for instance, food and fuel poverty, debt, bereavement, relationship breakdown, and sexual/domestic abuse. The accumulation of these factors then makes it difficult for people living in the most deprived areas to dedicate time and resources to protecting and managing their health.

That is not to say people are without the desire to make such changes; rather and as Elliott et al. (2015: 227) have commented, “what some professionals and/or researchers see as fatalism or a low locus of control are revealed as realistic assessments of the limited opportunities people have to control their lives”. Perhaps what is needed, then, is the approach of ‘empathetic ethnographers’ as suggested in findings from Garthwaite et al. (2016). In considering potential directions for future investigation, their participants were clear that health inequalities researchers need to interrogate what such inequalities mean in people's social worlds, as suggested by Scambler (2012: 144). This included prioritising listening to, and working to understand, the experiences of communities experiencing the brunt of health inequalities; again, especially important at a time of austerity.
